# A qualitative study of stakeholder and researcher perspectives of community engagement practices for HIV vaccine clinical trials in South Africa

**DOI:** 10.1002/jcop.22951

**Published:** 2022-11-07

**Authors:** Janan J. Dietrich, Jessica Munoz, Gugulethu Tshabalala, Lerato M. Makhale, Stefanie Hornschuh, Francisco Rentas, Mamakiri Mulaudzi, Fatima Laher, Michele P. Andrasik

**Affiliations:** ^1^ Perinatal HIV Research Unit (PHRU), School of Clinical Medicine, Faculty of Health Sciences University of the Witwatersrand Johannesburg South Africa; ^2^ Health Systems Research Unit South African Medical Research Council Cape Town South Africa; ^3^ African Social Sciences Unit of Research and Evaluation (ASSURE), Division of the Wits Health Consortium University of the Witwatersrand Johannesburg South Africa; ^4^ Ohio State University College of Medicine Columbus Ohio USA; ^5^ Department of Emergency Medicine Loma Linda University Medical Center Loma Linda California USA; ^6^ Wits Reproductive Health and HIV Institute (Wits RHI), Faculty of Health Sciences University of the Witwatersrand Johannesburg South Africa; ^7^ Vaccine and Infectious Disease Division Fred Hutchinson Cancer Center Seattle Washington USA

**Keywords:** CAB, Community Advisory Boards, community engagement, HIV, HVTN, South Africa, vaccine trials

## Abstract

Community engagement increases community trust of research and improves trial participation. However, there is limited documented appraisal of community engagement practices. Several HIV vaccine efficacy trials have been conducted in South Africa, the country most affected by HIV, predominantly in collaboration with the HIV Vaccine Trials Network (HVTN). We explored stakeholder and researcher perspectives of the HVTN community engagement practices used in the Gauteng province of South Africa. In 2017, we conducted a qualitative study. Using semi‐structured interview guides, we facilitated two group discussions with Community Advisory Board (CAB) members (*n* = 13), and 14 in‐depth interviews with HVTN‐affiliated employees (*n* = 8 in South Africa and *n* = 6 in the USA). Group discussions and in‐depth interviews were audio‐recorded, transcribed verbatim, translated into English, and coded using NVIVO 12 Plus software for thematic data analysis. Overall, median age of study participants was 22 (interquartile range 32–54) years, and 74% (*n* = 20) were female. Three main themes about community engagement emerged: (i) community engagement as an ongoing iterative relationship between researchers and community; (ii) methods of community engagement, encompassing community education by linking with external stakeholders and through awareness campaigns by pamphlet distribution and mass events, working with communities to develop recruitment messages, and working with CAB as a link to communities; and (iii) strategies to improve community engagement, for example, using simple language, linking with religious leaders and traditional healers, and communicating via conventional (newspapers, television, and radio) and social (videos and listicles) media. Our data indicate ways for researchers to improve relationships with community by understanding local needs, strengthening collaborations, and tailoring communication strategies. In this regard, CABs signify critical linkages between researchers and communities. CABs can relay relevant health research needs, advise on the creation of suitable materials, and link researchers more effectively with community leaders and media.

## 1 INTRODUCTION

In the context of research, community engagement is central to the ethical conduct of biomedical HIV prevention trials (Kagee et al., 2020). Community engagement, the interaction between researchers and communities in which research is conducted, is most successful as a dynamic partnership that empowers collaborative decision‐making for mutual good (Musesengwa & Chimbari, 2017). The World Health Organization and the Joint United Nations Programme on HIV/AIDS (UNAIDS) provide specific guidance on the role of ensuring transparent and meaningful community partnerships between research trial sites and stakeholders (UNAIDS & WHO, 2021). The process of community engagement involves establishing and maintaining relationships with diverse organizations, stakeholders, and individuals; understanding community needs, and regularly communicating research information to stakeholders and community members (Cunningham‐Erves et al., 2020). These activities are typically tailored to location, population, and social and structural factors (Kagee et al., 2020). Guidelines such as Good Participatory Practice (GPP), the South African Guidelines for Community Advisory Groups, and the Recommendations for Community Engagement in HIV/AIDS Research have been developed to facilitate optimal stakeholder involvement for the ethical conduct of biomedical HIV prevention trials (Gengiah et al., 2017; NHREC, 2020; Office of HIV/AIDS Network Coordination, 2014; UNAIDS, 2011). South Africa continues to bear a high burden of HIV, with almost 8 million prevalent cases in 2021 (UNAIDS, 2022). As such, HIV prevention organizations conduct both early phase and efficacy trials in the country. Most HIV vaccine clinical trials in South Africa have been conducted in collaboration with the United States (US)‐based HIV Vaccine Trials Network (HVTN; The Office of HIV/AIDS Clinical Trials Networks, 2014). The HVTN model of community engagement is rooted in GPP for biomedical HIV prevention trials and is informed by community‐based participatory research approaches that include community input throughout the research process, from trial design and implementation to results dissemination (Broder et al., 2020). A review on stakeholder engagement in HIV vaccine clinical trials found that predominant engagement is practiced during stages of protocol development and trial recruitment strategies and less frequent on posttrial results and dissemination (Day et al., 2018). Effective community engagement is associated with increased trial participation (Karim et al., 2017; Newman & Rubincam, 2014). Volunteer‐based Community Advisory Boards (CABs) are essential to these efforts. CABs exist at all HVTN clinical research sites, and CABs from each country with HVTN‐affiliated sites nominate a representative to join the Global CAB (Robinson et al., 2014).

### 1.1 HVTN community engagement model

The HVTN provides training on its community engagement model to all affiliated researchers. This model is described as three inter‐linked processes: community education (to set the foundation for awareness, literacy, and support of HIV prevention research), recruitment (to engage and support clinical trial participation), and retention (to support continued study engagement by the local community and a good trial experience by participants) (Broder et al., 2020). The philosophy behind the training is that community engagement is a collective responsibility shared by investigators, community staff, clinicians, and CAB members.

HVTN trial sites are expected to support their local CABs as part of their continuous and active partnership between clinical research sites and the community (HIV Vaccine Trials Network, 2020b). HVTN trial sites facilitate monthly CAB meetings and reimburse CAB member travel to site meetings, training workshops, and Network meetings. The goals of the CAB are to advise researchers about community norms, expectations, and concerns regarding research; disseminate information to the community about HIV and HIV research activities; assist in creating a supportive community environment for trial participants and their families; advise researchers on the design of clinical trials; review and revise participant information leaflets and informed consent documents, and advise potential and current trial participants of their rights during research participation. The HVTN involves CAB members during the review of study concepts and protocol drafts, during community education and results in dissemination activities, and throughout trial implementation (Kublin et al., 2012).

Effective community engagement, including facilitating ongoing discourse between researchers and community stakeholders, has been shown to enhance community trust in research in vulnerable communities and reduce misconceptions about the research being conducted (Newman et al., 2015). One study at South African HIV vaccine trial sites found that key challenges to community engagement included community mistrust about why particular communities were selected for research, the storage of biological samples, vaccination, and vaccine‐induced sero‐positivity (Thabethe et al., 2018). A study through the HVTN developed a multi‐part community engagement approach to support the inclusion of Black, Indigenous, and People of Color within COVID‐19 vaccine trials in the US. An intentional and a robust community engagement strategy is needed to ensure equitable and meaningful trial inclusion of underrepresented communities, while building trustworthy partnerships between the communities and institutions (M. P. Andrasik, Broder, et al., 2021). In the recent efforts of the COVID‐19 trials through the COVID‐19 Prevention Network (CoVPN), the strong and solid foundation of the community engagement framework and infrastructure through the HVTN allowed to readily and successfully engage priority communities and address trial‐related challenges in the United States (M. Andrasik, Rentas, et al., 2021). Despite the importance and successes of community engagement, efforts to document and appraise those practices have been limited, especially within resource‐limited research settings (UNAIDS, 2011). This study aimed to explore stakeholder and researcher perceptions of community engagement practices utilized by the HVTN in South Africa.

## 2 METHODS

We conducted a qualitative study from June 2017 to July 2017 utilizing group discussions (GDs) and in‐depth interviews (IDIs). Table 1 summarizes the GD and IDI methodologies. GDs were designed to draw upon the shared experiences of CAB members by exploring effective strategies, challenges, and lessons learned at trial sites. Before data collection start, the study was presented to interested leadership and staff members of the HVTN and the respective CABs within the South African‐based clinical trial sites.

**Table 1:** Methodologies of group discussions (GDs) and in‐depth interviews (IDIs)

	GDs	IDIs
Number conducted	Two GDs (a limit of 10)	Fourteen IDIs
Eligible participants	CAB members at one of two pre‐specified HVTN‐affiliated trial sites for at least 1 year	Employees who conducted HVTN community engagement activities for at least 1 year
Sampling strategies	Convenience	Snowballing and purposive
Method of approach	Face‐to‐face and telephonic	Email
Sample (participants)	Site 1 = 6 participants	South Africa = 8
	Site 2 = 7 participants	United States = 6
Nonparticipation	Approximately 35 CAB members approached, 13 participated	22 participants approached,
		14 interviewed
Setting of data collection	Two HVTN‐affiliated trial sites in South Africa	Telephonic = 10
		In‐person = 4
Interview guide	Semi‐structured	Semi‐structured
Mean duration (min)	174	42

Abbreviations: CAB, Community Advisory Board; HVTN, HIV Vaccine Trials Network.

### 2.1 Recruitment procedures

Convenience sampling was used to select participants for GDs. A qualitative researcher explained the study during a routine CAB meeting at trial sites. Community liaison officers (staff members employed by trial sites to communicate with CABs) then provided contact details of potentially interested and eligible CAB members to the qualitative researcher, who telephonically confirmed their interest and scheduled a GD. Two GDs were conducted, one at each of the pre‐identified trial sites in the Gauteng province of South Africa, which had an HIV prevalence of 17.6% at the time of our study (Simbayi et al., 2019).

IDIs, which used purposive and snowball sampling, sought the perspectives and experiences of individuals employed to engage community members about HVTN trials. We invited HVTN employees by email to participate, and, in South Africa, employees could recommend individuals who were involved in community engagement. Participants could choose a convenient time for their IDIs, and whether it was conducted in‐person or telephonic.

### 2.2 Participant selection and inclusion criteria

GD participants comprised local South African CAB members who were affiliated with two pre‐specified HVTN clinical trial sites in Gauteng province for at least 1 year.

IDI participants included HVTN staff from South Africa and globally who had been employed for at least 1 year with an active role in community engagement for clinical trials with HIV‐negative adults who are at high risk for HIV.

### 2.3 Data collection procedures

On the day of the GD or IDI, participants first provided voluntary written consent and then completed a demographic questionnaire before participation in the GD/IDI. For confidentiality, the researchers asked participants to use a pseudonym during discussions and interviews.

### 2.4 Demographic questionnaire

All participants completed a brief socio‐demographic questionnaire that assessed quantitative variables (Table 3).

An English‐speaking, Hispanic medical student from the United States of America was trained in qualitative data collection to conduct the GDs and IDIs. The GD guide was designed to elicit discussion on CAB members shared experience of community engagement (Table 2). The IDI guide was designed to prompt participants to discuss community engagement definitions and activities, factors facilitating optimal community engagement, community outreach best practices, community discourse regarding HIV and/or HIV vaccine trials, HIV messaging, HIV education strategies, and best practices for gaining community support (Table 2).

**Table 2:** Summary of data collection process

Participant self‐reported questionnaire	Age, gender, country, primary home language, highest level of education, current job
Topics in the GD guide	1. What if you could create a message for your community about HIV vaccine trials? 2. What would be the best way to explain and gain the support of your community for HIV vaccine trials? 3. How could we best educate your community about HIV vaccine trials? 4. What leaders in your community have the strongest impact on your actions and beliefs?
Topics in the IDI Guide	1. How do you think HVTN can improve HIV vaccine trial community support? − Probe: What do you think works best in terms of engaging communities? 2. How can Community Advisory Boards improve community outreach? − Probe: What if you could create a message for your community about HIV vaccine trials? 3. What would be the best way to explain and gain the support of your community for HIV vaccine trials? 4. How could we best educate your community about HIV vaccine trials?

Abbreviations: GD, group discussion; HVTN, HIV Vaccine Trials Network; IDI, in‐depth interview.

### 2.5 Data analysis

The audio recordings of GDs and IDIs were transcribed verbatim and translated into English for thematic data analysis (Braun & Clarke, 2006). A primary analyst (GT) achieved data immersion by reading the transcripts of the first two GDs and two IDIs to understand and familiarize herself with the data. All four transcripts were coded using a line‐by‐line technique to assign codes to text (Braun & Clarke, 2006). Two additional coders (JM and FR) then independently coded the data using the same line‐by‐line technique. All three coders then met to discuss their identified codes, where similarities and differences were discussed. For any differences identified a consensus was reached to agree on appropriate codes. Codes, which emerged deductively using the interview guides and inductively using the emerging data from the transcripts were captured with NVIVO 12 Plus data analysis software and a comprehensive codebook was developed. The codebook was shared and reviewed by the study team. All remaining transcripts were then coded in NVIVO 12 Plus. The codes were then sorted, collated, and categorized into themes and subthemes.

### 2.6 Ethical considerations

The study protocol was approved by the University of the Witwatersrand Human Research Ethics Committee (Medical). All participants provided voluntary written informed consent before study procedures. Participants of GDs were reimbursed ZAR100 (~US$7.65 at the time of study conduct) for transport costs. Participants of IDIs were not reimbursed because no transport costs were incurred. Principal investigators of both clinical research sites provided permission for the study, for CAB members to be approached, and for the GDs to take place in a confidential room at the HVTN sites.

## 3 RESULTS

### 3.1 Participant demographics

Across IDI and GD participants, the median age was 22 (interquartile range [IQR]: 32–54) years, 74% (*n* = 20) were female, 26% (*n* = 7) spoke English as their primary home language, and 52% (*n* = 14) had completed training after high school. Of IDI participants, eight were community engagement staff, three were research assistants, two were recruiters, and one was a socio‐behavioral science project coordinator (Table 3). Participants in the IDI were affiliated by work to HVTN studies and thus were involved in community engagement activities. Participants in the GDs were CAB members, who functioned as a bridge between HVTN researchers and community members and thus were involved in community engagement activities.

**Table 3:** Participant demographics

Variables		Total *N* = 27
**Age**	Median [IQR]	22 [32–54]
**Sex**	Male	7 (26%)
	Female	20 (74%)
**Country**	South Africa (SA)	21 (78%)
	United States of America (USA)	6 (22%)
**Race** a	Black	22 (81%)
	Caucasian/white	3 (11%)
	Latino	1 (4%)
**Primary home language**	English	7 (36%)
	Spanish	1 (4%)
	isiZulu	6 (22%)
	Sesotho	1 (4%)
	Isi Xhosa	3 (11%)
	isiNdebele	3 (11%)
	North Sotho	3 (11%)
	Sepedi	1 (4%)
	Xitsonga	1 (4%)
	Afrikaans	1 (4%)
**Highest level of education**	Incomplete primary school	1 (4%)
	Completed primary school	1 (4%)
	Incomplete high school	/
	Complete high school	7 (26%)
	Incomplete post‐high school training	4 (15%)
	complete post‐high school training	14 (52%)
**Role**	CAB member	13 (48%)
	Community engagement staff	8 (30%)
	Research Assistant	3 (11%)
	Recruiters	2 (7%)
	Socio‐behavioral science project coordinator	1 (4%)

Abbreviation: IQR, interquartile range.

^a^ Missing data – race (*n* = 1).

### 3.2 Emerging themes

#### 3.2.1 Theme 1: Community engagement as an ongoing iterative relationship between the researchers and the community

Key subthemes included (a) Community involvement in research design and implementation; (b) Community awareness of new and ongoing research developments; and c) Building and fostering relationships.

(a) Community involvement in research design and implementation.
  Some participants from the IDIs described community engagement in HIV vaccine trials as an ongoing iterative process of involving the community in the design and implementation of a research concept.
*“Community Engagement is a way of bringing in the community to be partners in the design and implementation of a research concept or a research study and it is an ongoing process a process that should take place well in advance before a concept is designed and through the selection of sites until the end of the research and that is only able to happen when you involve the community through your engagement mechanisms.” (IDI‐004‐SA)*
*I think it's involving the community in all aspects of our research. whether that is in protocol development, whether that is in, um, education, so educating the rest of the population – educating them and working with them to figure out what this education should look like.” (IDI‐022‐USA)*
*“Community engagement would be … engaging the community in the work that we do, asking for their guidance and what they think of the research that we do, and also, encouraging them to participate in the work that we do.” (IDI‐005‐SA)*
(b) Community awareness of new and ongoing research developments.
  A few IDI participants highlighted that community engagement involves the responsibility to keep the community engaged and informed about new developments in the research and to ensure that the community is aware of the aims of the research specific to their community and any major developments.
*“I would say community engagement is a multi‐faceted attempt to be connected to both people that could potentially enroll in your studies, and people that are in the community in general. It's a mix of education, It's a mix of branding, It's a mix of social media.” (IDI‐021‐USA)*
*“I think it is to maintain community updates on HIV research in general and what is happening within the field of HIV research as well as informing specific communities about specific trial, ensuring that they receive results or outcomes trials that we also hear from communities about their concern or answer questions or hear their opinions about the research trials that are being conducted.” (IDI‐023‐USA)*
*“Ensure that that the communities at large are aware of it [research]; the type of research being conducted within their geographic area; what is the intent or the aim of the research trials or what are the aims or what is the outcome that we are looking for. Why this particular research trial being conducted.” (IDI‐023‐USA)*
(c) Building and fostering relationships.
  Community engagement was also perceived by a few IDI participants to be an important process in building a relationship between the researchers and the communities that they were involved in. They emphasized the importance of ensuring that long‐lasting relationships are fostered to enhance collaboration and future engagements.
*“It's also building relationships and trying to sustain those relationships, and sort of navigating the best ways to collaborate. It's an ongoing constant effort to find the best ways to reach people and that's never a one‐off answer. There's a different answer for every person so it's really like a constant sort of space to innovate ways of fostering connections.” (IDI‐021‐USA)*
*“It's a mix of education, branding, social media. It's also building relationships and trying to sustain those relationships, and sort of navigating the best ways to collaborate. It's an ongoing constant effort to find the best ways to reach people and that's never a one‐off answer.” (IDI‐021‐USA)*
*“Community engagement, actually, is, um, a relationship that one should have with the community at large, and having to tell them the (.) purpose of the research studies that we do in simple and understandable language of the actual – able to engage with the community.” (IDI‐024‐SA)*
  Two IDI participants perceived that it was important for researchers to understand the needs of and collaborate with the community. Community engagement was viewed as a process in which researchers sought guidance from the community.
*“Community engagement is being a part of the community, being involved in the community, knowing what the community wants and understanding their needs basically more than their wants and being a part of them more than anything.” (IDI‐013‐SA)*
*“Community engagement would be as a clinical site, engaging the community in the work that we do, asking for their guidance and what they think of the research that we do, and also, encouraging them to participate in the work that we do.” (IDI‐005‐SA)*

#### 3.2.2 Theme 2: Methods of community engagement

Participants of GDs and IDIs stated the following community engagement methods were important: (a) educating people in the community about research studies by linking with external stakeholders, (b) educating the community through awareness campaigns, (c) working with communities to develop recruitment messages, and (d) working with CABs as the link to the communities.

(a) Educating people in the community about research studies by linking with external stakeholders.
  Most participants stated that community education helps build support for a clinical trial and ensures trial participants are knowledgeable and informed about research studies currently being conducted at a clinical research site. Participants perceived that successful community engagement was contingent on collaboration and partnership with community stakeholders, who are trusted, respected, and recognized members of that community. Furthermore, participants indicated the necessity of research organizations training their staff, so they feel empowered to dispel myths and misconceptions in the communities where they work and live. Participants in both the IDIs and GDs recommended educating the community through community meetings and awareness campaigns and inviting participants from previous clinical trials to share their experiences.
*“Having consultations where the community was involved … in letting us know one of the myths and misconceptions that people have about HIV and what are the needs. We're interacting with the community to determine what their needs are and what they think needs to be covered in the com [community] – what topics need to be covered in the community around HIV and HIV vaccine trials.” (IDI‐022‐USA)*
*“The one other thing that they [researchers] can look into is to emphasize more on outreach activities because for the community to buy into what the researchers are doing it takes time, sometimes it takes effort. But sometimes by doing, some people learn by doing, when we come to them, do something in front of them, explain what we do and why the reason for doing that, they [people in the community] easily buy into whatever we do or we say. So, I think being more interactive with the people will make a difference and will get the message across better.” (GD‐001‐CAB Members)*
*“I think it would really be to do what is being currently done where we have we do education at multiple levels; we have mass targeted education that just highlights vaccine research. Education that targets specific groups of people could be I think one thing that can be done better is to have people in each community who would be brought in especially people who are not employed who would be brought in and become some form of ambassadors who really are taken through a program of HIV vaccine research literacy. So that they are able to send out information into the community into the broader community and this group is kept as a constant group that you know is incentivized to an extent but they don't have a specific role; they are just given information and there are mechanisms of having them give out information to different groups.” (IDI‐004‐SA)*
  A few IDI participants regarded collaboration with external stakeholders as a crucial component in community engagement. It was perceived that the purpose of a partnership between research sites and community stakeholders was to learn about common misconceptions about HIV vaccines, assist researchers in sharing correct information, and create links with marginalized populations.
*“We go outside with our pamphlets, and educate people, and we have external stakeholders that invite us to the events. So, we get there, we have a platform, we talk about all our studies, addressing myths, misconceptions, and false statements.” (IDI‐005‐SA)*
*“I think community partnerships with other organisations [work with other organisations] are really important for education because community organizations outside of the trial, of the clinical research site broadens the reach of the site quickly, and oftentimes, if you are strategic enough about the organizations that you partner with, then it can help you reach people that you would have no other way to reach.” (IDI‐002‐USA)*
(b) Educating the community through awareness campaigns.
  Participants valued awareness campaigns as a method of raising awareness about HIV vaccines and trials to a large audience in a community at once. Most participants thought that awareness campaigns are an effective strategy of educating and motivating people to find out more about HIV vaccines.
*“I think that awareness is much better because no one can understand what you are saying without teaching or telling him or her. It's our work and now when we are discussing here, I can see that there is more work to do than awareness. Firstly, awareness; education; campaigning whereby they [community] understand what we are doing.” (GD‐002‐CAB Members)*
*“With the community engagement, being a part of the community, or being involved in the community, it involves us doing education sessions about the HVTN and speak about HVTN is our HIV Vaccines Awareness Day. We host it at different parts of the community and we invite our various stakeholders and the community members and we involve them way before the day of the event.” (IDI‐013‐SA)*
*“So basically, we have what we call community engagement board – the CAB members that liaise, with the community, between the research site and the community together and they also host events. We sometimes do awareness campaigns in the community just to engage with the community and inform them about the studies that we do. (IDI‐024‐SA)*
(c) Working with communities to develop recruitment messages.
  Most IDI participants stated the importance of different messaging considerations to deliver accurate information. Messages should be adequately tailored, for example toward education level and language, to recruit participants into clinical trials.
*“Conducting a series of small focus groups with people that might be eligible. The goal of the focus groups is really to look at what messages and what images are best for our recruitment campaign and it's a chance for us to get input on the level of sort of educational background [of] people, need for ads or what's appealing. That's one way of engaging communities.” (IDI‐021‐USA)*
*“I think having information that states exactly what is it that we [researchers] do, and also, it should be in a language that they can understand best. And, that information should come from someone who once maybe participated in the [HIV vaccine] trial, or maybe someone who lives in the community that maybe they look up to, maybe like the CAB members.” (IDI‐025‐SA)*
(d) Working with CABs as the link to communities.
  Most IDI participants stressed the important role CABs play by communicating and educating the community on HIV vaccine trials. They further stated that communication and CAB involvement in community engagement was regarded as crucial for gaining trust, creating a safe and comfortable space to share and express themselves, building a relationship with the community, and understanding the research. It was perceived that effective communication can be achieved when community members attend general meetings, host awareness campaigns, and engage with CAB members.
*“Before they [HVTN] even begin the protocol, they involve the CAB members, which are part of the community. They involve them in the drafting and putting together of the consent forms… So, it's not something that you just see happening in your community.” (IDI‐013‐SA)*
*“We sometimes do awareness campaigns in the community just to engage with the community and inform them about the studies that we do. Those are the kind of community engagement events that we do.” (IDI‐024‐SA)*

#### 3.2.3 Theme 3: Strategies to improve community engagement

Key subthemes included (a) using simplified language to explain research terms; (b) linking with religious leaders and traditional healers; and (c) using media as a method of sharing and educating the community about HIV research.

(a) Simple terminology.
  A few IDI participants indicated that when working with the community, the clinical research site should use simplified language to explain research processes and information. These include information in consent forms and through community engagement efforts.
*“So, like for one, with our consent forms, we know we experienced – there's some words that are just vague, so we try to break them down in simple English. Like, instead of saying experimental we'd use “testing.” (IDI‐005‐SA)*
(b) Linking with religious and traditional healers.
  Some participants stated the importance of a strategy to preserve community rights and decision‐making power. In addition, the participants expressed how communities are diverse and multidimensional, with multiple religious and traditional beliefs. They indicated that partnering with various religious organisations could enhance education and social support.
*“To work with the community sometimes you must have a strategy – it is their right and you cannot force them. If I believe in traditional healer or in Western medicine, you cannot force me [to take either]. So you must educate me and tell them that even if the have beliefs and rights – you must tell them that rights come with responsibilities. I am HIV‐positive and I believe in God – but I must be responsible for my body. This is a human need, and I know that my soul needs God but the flesh needs medication. So we must educate them – teach.” (GD‐002‐CAB Members)*
*“I think that the other portion of it is having the consistent buy‐in from community stakeholders who would actually want to continue the conversation. So, thinking of ways, like, how do we support organizations, have these conversations, whether it's a church or a dance studio.” (IDI‐021‐SA)*
(c) Media.
  Some participants provided suggestions to incorporate the media for information sharing, including using traditional print media as well as social media. One participant said:
*“I think just being mindful of things, like maybe recording things, thinking of ways, how to promote it in videos on Facebook. They could do, like, more of – the Buzzfeed thing where it's like a short video with captions. There could be things like what they call listicles, where it's like the article's like. “Ten Things You Need to Know About Vaccines.” Or “Ten Reasons Why–” “Ten Things About Medical Mistrust” or “Ten Medical Injustices,” and then also, “Ten Ways That Researchers Are Trying to Protect Participants Now.” (IDI‐021‐USA)*
*“So, if HVTN sometime have adverts maybe once or so a day on radio or TV … The newspapers if they can even have one page in each of the newspapers just to tell the people that we are here as HVTN and we do this and this.” (GD‐002‐CAB Members)*
*“I think that we need to always be transparent and share information. [for example] pre‐launching a research trial during the enrolment phase of the trial and the results!” (IDI‐023‐USA)*

## 4 DISCUSSION

This qualitative study is to our knowledge one of the first to document the expectation of CAB members and community engagement employees on the HVTN's community engagement practices in the South African setting. Community engagement is an ongoing iterative relationship between researchers and community from trial design to implementation, posttrial, and results dissemination. Community engagement is a critical ethical protection for health research and is globally encouraged by international ethics guidelines (Council for International Organizations of Medical Sciences (CIOMS), 2016). Our data reemphasize prominent and accepted methods of community engagement in South Africa and provide suggestions for strategies to improve community engagement for clinical trials.

Methods for nurturing community engagement for HIV vaccine clinical trials included fostering partnerships with trusted and respected community and external stakeholders, capacity building of research staff and community representatives, education through community meetings and awareness campaigns and working with the CAB as the direct community link. These methods form part of the principles set out for effectively engaging the communities in HIV/AIDS research by a global group of community representatives affiliated with HIV/AIDS clinical trials networks funded by the National Institutes of Health (NIH; Office of HIV/AIDS Network Coordination, 2014). These principles include to identify the needs of and learn about the community, develop cultural competency, foster transparency, build partnerships and trust, promote capacity building, and maintain long‐term commitment. Even though the current engagement processes practice many of the principles already, reviewing and operationalizing the foundational principles based on individual community needs may enhance community empowerment, investment, and support of the research being conducted.

Community engagement employees of the HVTN recommended training research staff as well as linkage to external stakeholders to empower and educate to ensure that the correct knowledge around HIV and vaccine‐related topics is shared and to address myths and misconceptions to communities and trial participants. Empowering site staff and CAB members through ongoing capacity building, access to resources, and active listening to community voices, build trustworthiness and partnerships, important ingredients in the successful execution of a trial (Quinn & Andrasik, 2021; Thabethe et al., 2018).

HVTN employees further emphasized on the need to simplify the English language when sharing research information in consent forms and local community engagement efforts. Many clinical trial consent forms are lengthy and often include complex language and concepts (Fischer et al., 2021). Current South African ethics in health research guidelines recommend that the language used in consent documents should be no more than at an eighth‐grade reading level (Department of Health, 2015). The same readability level expectation is given to translated documents into the local South African languages (e.g., isiZulu, isiXhosa, and Sesotho), as for many participants the English language is a second or third language. Therefore, continued attention should be given to ensure that review in collaboration with the local community is sought to ensure that the suggested literacy levels are met for all study and community engagement materials. In addition, staff training on consent content is imperative to elicit competing representation of complex trial concepts (Rautenbach et al., 2015).

Performed in a time before the COVID‐19 pandemic, this study highlights the value of in‐person community engagement strategies. South African participants emphasized creating and maintaining relationships through mass events and in‐person meetings with stakeholders such as community‐based organizations, and key opinion leaders such as religious leaders and traditional healers. The authors note that although in‐person engagement strategies have been heavily relied upon historically, these same strategies may be ill‐advised due to public health concerns during an airborne viral pandemic. There are few immediately practicable replacements to in‐person community engagement; three such suggestions from this study include television, newspapers, and radio. Our study participants in the US suggested adopting social media platforms for community engagement. The Pennsylvania HIV Planning Group in the US implemented remote community engagement strategies, including video conferencing tutorials, online meetings, and hybrid outreach methods, which resulted in equal and even more attendance compared to the pre‐pandemic meetings (Givens et al., 2021). However, in many settings within South Africa, most people do not have access to a computer, mobile phone data is costly and access to electricity is intermittent. Even though online and remote community engagement strategies have shown to be effective during the pandemic and have benefits (Manikam et al., 2021), it is important to employ hybrid approaches to ensure accessibility in engagement for marginalized populations, geographically isolated and those with limited access to online options (Carson et al., 2020; Givens et al., 2021; Newman et al., 2021). This may include targeted one‐on one outreach, printed resources and materials, audio and video recordings, local community media, and engagement with local community leaders (Carson et al., 2020).

The historical preference for and reliance upon in‐person community engagement in South Africa, and technological access challenges with alternatives, has implications for not only HIV vaccine research in the long term, but immediately for COVID‐19 vaccines. Undeniably, for a trial to test a vaccine for safety, immunogenicity and efficacy, community members need to be willing to participate (Newman et al., 2007). Such success depends on communities being educated about the research before and throughout implementation (CTSA Community Engagement Key Function Committee, 2011). In addition, an approved vaccine requires community involvement to facilitate the success of its implementation and uptake. In this way, community engagement is a precursor to being informed: a critical component of the consent process (Newman et al., 2015).

Although continuous engagement is described as the best practice (Cramer et al., 2018), the COVID‐19 pandemic poses major impediments to maintaining in‐person relationships with stakeholders and communities, a strategy that has been the historical foundation of community engagement. Community engagement is perceived as critical to build trust and provide communities with the tools to make informed decisions about research. The findings of this study allude to the existing community engagement infrastructure across the different HVTN sites that has been built in the past two decades with the foundation of the HVTN's community engagement framework (Andrasik et al., 2020; Broder et al., 2020). This study reemphasizes the importance of the respective CABs and their involvement in the continued efforts of building and maintaining trusted community partnerships. This is supported by research conducted through the CoVPN, where HVTN's community engagement framework infrastructure provided a solid foundation to readily and effectively engage communities and address trial challenges (M. Andrasik, Broder, et al., 2021). Our study further supports the requirement for researchers to engage communities continuously, for example by working with CABs to understand community needs, and by collaborating with community stakeholders to deliver tailored messages to address recruitment needs, trial challenges and to educate on myths and misconceptions.

This study recommended that enhancing relationships with traditional and religious stakeholders to strengthen community engagement will ensure to address the multidimensional needs of the communities. Their involvement will inform and take into account the diverse religious and traditional beliefs when providing social support and education. A study conducted with religious leaders in Soweto found that most did not feel equipped and skilled enough to address and educate on issues relating to HIV/AIDS even though they felt the need to do so (Alio et al., 2019). This could translate into traditional and religious stakeholders being more connected to the CABs of the trial site and involved in community‐engaged activities to receive the appropriate support and training needed.

Our study demonstrates that the community engagement methods used in South Africa revolve predominantly and preferably around in‐person relationship building, for example, through mass events and physical attendance at meetings. It is possible therefore that safety considerations imposed by the COVID‐19 pandemic, especially on a continent where vaccination coverage is lagging behind, may threaten the continuation of effective community engagement. Participants from this study recommended engaging with the community through media, including social media platforms in addition to in‐person engagement. To mitigate potential challenges posed by the COVID‐19 pandemic, future investigation is needed to understand the potential of developing and utilizing mobile health and hybrid strategies in supporting the ongoing and successful community engagement efforts and reaching the desired study populations remotely.

### 4.1 Limitations

Our study does have some limitations. First, the study conducted GDs with CAB members at two South African clinical research sites only, which does limit the generalizability of the findings to other sites. CAB members and HVTN employees participating in the data collection might have been subject to social desirability bias and might not have been too critical of the HVTN. Therefore, challenges and issues towards community engagement might have not been raised by the participants. The interviews were conducted by a medical student from the US who might not have been aware of the cultural aspects in South Africa. However, for the GDs the student was supported by a local Black researcher. Participants did not review transcripts and the authors' interpretations, but findings were shared at an international HVTN meeting with representation of international and local South African CABs, community engagement stakeholders and staff, and HVTN leadership.

### 4.2 Implications

This study has implications for community engagement practices and processes for HIV vaccine trials research. Designing of community engagement materials, awareness campaigns and consent forms for relevant communities and trial participants should ensure to simplify the English language to not more than an eighth‐grade reading level and provision of translation to the main local languages. This will ensure that critical marginalized populations with low literacy levels can be reached and involved. In addition, research staff need to receive continued and adequate training on how to disseminate and address relevant information appropriately to the local communities and to conduct the consent process meaningful, while being able to address language barriers for complex content. The simplified language and translated materials should be reviewed by CAB members and potential participants to ensure adequate levels of literacy. Furthermore, trial teams should ensure that staff from the local communities are employed that speak the main local languages. Currently, community engagement practices within the HVTN are established, reviewed, and documented (HIV Vaccine Trials Network, 2020a, 2020b). However, we would recommend that community engagement practices and processes be formally evaluated for empirically peer‐reviewed publications.

## 5 CONCLUSION

Our data indicate ways for researchers to engage communities by understanding local needs, strengthening collaborations, and tailoring communication strategies. We present practical implications and recommendations for community engagement for HIV vaccine clinical trials. CABs continue to be critical linkages between researchers and communities. CABs can relay relevant health research needs, advise on the creation of suitable materials, and link researchers more effectively with community leaders and media.

## AUTHORS CONTRIBUTION

JJD, JM, MPA conceived the idea. JJD, JM, SH, MA contributed to study design, including the measures design. Data collection were performed by JM and LMM. JM, FR and GT contributed to data analysis. JJD wrote the first draft of the manuscript. FL contributed to manuscript writing and edited the manuscript with important intellectual content. MM and SH contributed to manuscript writing and editing. At the time of the study, all authors were employed as researchers except JM was a medical student.

## CONFLICT OF INTEREST

FL is a clinical research site leader of an HVTN‐affiliated vaccine trial site, and conflict of interest was minimized by ensuring she did not conduct the data collection or data analysis.

### PEER REVIEW

The peer review history for this article is available at https://publons.com/publon/10.1002/jcop.22951

## Data Availability

Data that support the findings of this study are available from JJD, but data are not publicly available owing to the need to protect participant confidentiality.
